# Impact of Pharmacists’ audit on improving the quality of prescription of dabigatran etexilate methanesulfonate: a retrospective study

**DOI:** 10.1186/s40780-017-0077-8

**Published:** 2017-01-17

**Authors:** Teppei Shimizu, Yoshio Momose, Ryuichi Ogawa, Masahiro Takahashi, Hirotoshi Echizen

**Affiliations:** 1 Department of Hospital Pharmacy, Kitahara International Hospital, 1-7-23 Owada-machi, Hachioji, Tokyo 192-0045 Japan; 2 Departments of Neurology, Kitahara International Hospital, 1-7-23 Owada-machi, Hachioji, Tokyo 192-0045 Japan; 3 Department of Pharmacotherapy, Meiji Pharmaceutical University, 2-522-1 Noshio, Kiyose, Tokyo 204-8588 Japan

**Keywords:** Hospital pharmacists, Prescription audit, Dabigatran, Electronic medical records, Prescribing information

## Abstract

**Background:**

Appropriate prescription of dabigatran etexilate methanesulfonate (JAN) is more complicated than assumed, because there are totally 10 items of contraindications and instructions for dosage reduction depending on patients’ characteristics. We aimed to study whether the routine audit of first-time prescriptions of dabigatran performed by pharmacists is effective in improving the quality of prescription.

**Methods:**

A retrospective re-audit was performed on all the prescriptions of dabigatran issued at Kitahara International Hospital, Tokyo between March 2011 and February 2014, by evaluating the prescriptions rigorously against the approved prescribing information of the drug. The original routine audit of the prescriptions for inpatients was performed by hospital pharmacists using electronic medical records (EMR), whereas the audit for ambulant patients receiving external prescriptions was performed by community pharmacists using information obtained mainly by questioning patients. The frequencies of inappropriate prescriptions detected by the re-audit in the two groups were compared.

**Results:**

Two hundred and twenty-eight patients (131 ambulant patients and 97 inpatients) were prescribed dabigatran for the first time during the study period. All patients met the approved indications. While 33% of the prescriptions for ambulant patients showed at least one violation of the approved usage, only 11% of the prescriptions for inpatients showed violations (*p* < 0.001). Two ambulant patients with creatinine clearance < 30 mL/min were dispensed dabigatran, whereas no such case was found among inpatients. A significantly greater proportion of ambulant patients aged ≥70 years showed violation of the instruction for dosage reduction compared to inpatients of the same age group (18 and 4%, respectively).

**Conclusion:**

The present study suggests that pharmacists may achieve better performance in auditing prescriptions of dabigatran when medical records are fully available than when information is available mainly by questioning patients. Further large-scale studies are required to clarify whether the audit of dabigatran prescriptions improves ultimate therapeutic outcomes or complications.

## Background

Dabigatran etexilate methanesulfonate (JAN) is the first direct oral anticoagulant (DOAC) launched in Japan for preventing thromboembolic complications in patients with non-valvular atrial fibrillation [1]. In contrast to warfarin, a vitamin K antagonist, the anticoagulation effect of dabigatran is not influenced by the variability of oral vitamin K intake, drug interaction with cytochrome P-450 (CYP) inhibitors, or genetic polymorphisms of CYP2C9 [2]. In addition, there is no need for routine monitoring with anticoagulation tests [1]. In this context, prescribing dabigatran may appear less complicated compared to warfarin. However, there are 6 items of contraindications and 4 items of instructions for dosage reduction in the prescribing information for dabigatran in Japan [1]. As a result, inappropriate prescriptions that violate the approved usage of the drug may not be uncommon, particularly in first-time prescriptions. To our knowledge, however, no attempts have been undertaken to study the frequency of inappropriate dabigatran prescriptions in Japan.

Previous studies performed in the USA and European countries have demonstrated that prescription audit by pharmacists may reduce the incidence of potentially inappropriate prescriptions and improve medication safety and patients’ adherence to pharmacotherapy, ultimately improving patients’ quality of life [3–5]. While many reports have documented the statistics of medication errors for any drugs in Japan and the impact of pharmacists’ intervention on preventing these errors [6, 7], it remains largely unclear whether prescription audit performed by pharmacists may reduce inappropriate prescriptions of DOACs. In our hospital, prescriptions of all drugs issued to inpatients are subject to pharmacists’ audit using electronic medical records (EMR), and any potential violation of prescribing instructions is fed back to the responsible physician, and revision is made whenever necessary. In contrast, external prescriptions are issued to ambulant patients according to the national policy of the separation of dispensary from medical practice. As a result, audit of dabigatran prescription is performed by community pharmacists using information obtained mainly by questioning the patients. Given this situation, we aimed to study the frequency of inappropriate dabigatran prescriptions, particularly for first-time prescriptions, and to study whether audit of prescriptions by pharmacists using EMR rather than questioning patients may improve the quality of dabigatran prescription, particularly at the initiation of anticoagulation therapy.

## Methods

The present study was a retrospective observational study performed at Kitahara International Hospital (KIH), Hachioji, Japan. We reviewed the medical records of all patients who started dabigatran therapy either as inpatient after admission to our hospital or as ambulant patient at the ambulant clinic of KIH between March 2011 and February 2014. We excluded patients who had been initiated dabigatran therapy elsewhere before they were referred to our hospital.

Before dabigatran was added to the drug formulary at KIH, pharmacists organized an educational meeting for all physicians who were going to prescribe the drug, regarding proper usage of the drug with respect to dosage individualization in patients with renal dysfunction and drug interaction with concomitantly administered drugs. Regarding the assessment of renal function, estimated glomerular filtration rate (eGFR) was automatically calculated according to the MDRD equation modified for Japanese by the Japanese Society of Nephrology with a built-in function of the EMR [8]. In contrast, physicians had to calculate creatinine clearance (CLcr) by themselves using the Cockcroft-Gault’s nomogram.

**Fig. 1:**
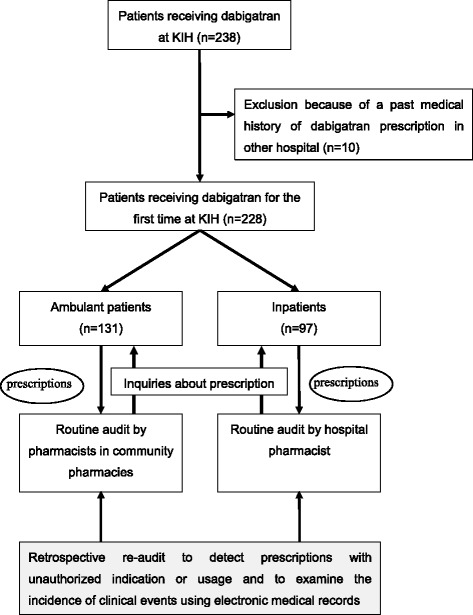
Design of the present study

Routine audit of dabigatran prescriptions for inpatients was performed by hospital pharmacists according to a checklist (Table 1). The checklist is compatible with the documents in the authorized prescribing information of Prazaxa® [1], except for the instructions for switching anticoagulation therapy from a parenteral anticoagulant (e.g., unfractionated heparin) to dabigatran. This is because parenteral anticoagulants are used only for inpatients. Audit of external prescriptions for ambulant patients was performed by community pharmacists (Fig. 1) mainly using information obtained by questioning patients. All audit inquiries regarding the original prescriptions were fed back to the responsible physicians and revisions were made where appropriate.

**Table 1:** An audit checklist for dabigatran prescription

Descriptions in prescribing information	Criteria
Indication	• Prevention of strokes and systemic thromboembolic complications in patients with non-valvular atrial fibrillation
Contraindications	• Severe renal dysfunction (CLcr < 30 mL/min or eGFR < 30 mL/min/1.73 m^2a^) • Active bleeding or hemorrhagic diathesis • Clinical complications associated with high-risk of bleeding (cerebral hemorrhage) within 6 months • Concomitant indwelling of spinal or epidural catheter • Concomitant oral administration of itraconazole • History of serious hypersensitivity reaction to Prazaxa®
Instruction of dose reduction (300 mg/day to 220 mg/day)	• Moderate renal dysfunction (CLcr 30–50 mL/min or eGFR 30–50 mL/min/1.73 m^2a^) • Concomitant oral administration of P-glycoprotein inhibitors^b^ • Age ≥ 70 years • Previous history of gastrointestinal bleeding
Instruction for timing of initiating dabigatran therapy after withdrawal of warfarin	• Dabigatran should be started after PT-INR decreases < 2.0

*CLcr* creatinine clearance

^a^According to the prescribing information of Prazaxa® [1] CLcr is recommended for evaluating renal function, but eGFR was used as an alternative when body weight was unavailable

^b^Verapamil, amiodarone, quinidine, tacrolimus, cyclosporine, ritonavir, nelfinavir, saquinavir and others

In the present study, we retrospectively re-audited all first-time dabigatran prescriptions that had passed the original routine audit performed and investigated the incidence of inappropriate prescriptions (Fig. 1). The incidence of inappropriate prescriptions outcome were compared between inpatients and ambulant patients. The protocol of the present study was reviewed and approved by the institutional review board of the KIH before the study was begun (#15-2014). The present study was conducted in compliance with the Declaration of Helsinki and the guideline for the collection, storage and handling of personal information of patients for healthcare professionals issued by the Ministry of Health, Labour and Welfare, Japan [9].

Renal function of patients was assessed by creatinine clearance (CLcr) according to the recommendation in the prescribing information, whenever possible. eGFR was used as an alternative when body weight of a patient was unavailable. For inpatients, we judged that physicians had considered renal function of patients when they measured serum creatinine concentrations within one week before they initiated dabigatran therapy. For ambulant patients, we adopted a less rigorous criterion. We judged that physicians had considered renal function when serum creatinine concentrations measured within three months were available at the initiation of dabigatran therapy. When no serum creatinine data were available within the respective periods, we judged that the drug was prescribed without considering renal function and therefore was inappropriate.

Using EMR, we checked all concomitant medications. We also investigated past medical history of gastrointestinal bleeding and upper gastrointestinal ulcer for each patient through EMR. While we did not adopt as an outcome measure, we searched for newly developed bleeding or thromboembolic event up to 1 year after the initiation of dabigatran therapy. When patients stopped taking dabigatran or visiting the ambulant clinic of KIH for any reason, their data collected until the last observation were included in the analysis. When patients were referred to the two KIH-affiliated ambulant clinics located in Hachioji city, their data collected in those clinics were included in the analysis. We evaluated the presence or absence of bleeding and its severity according to the criteria employed in the RE-LY study [10].

### Statistical analysis

Statistical analyses of the patients’ characteristics, numbers of concomitant medication and incidence of inappropriate prescriptions were performed using either Fisher’s exact test or the Student’s *t*-test for continuous variables and the Chi-squared test was for gender ratio. Odds ratios and 95% confidence intervals were calculated for the prevalence data. A p value less than 0.05 was considered to be statistically significant. The JMP software (version 11.0 SAS Institute Inc., NC, USA) was employed for statistical analyses.

## Results

We retrieved the demographic and clinical data of 137 ambulant patients and 101 inpatients who were prescribed dabigatran for the first time during the study period at KIH (Fig. 1). Six ambulant patients and 4 inpatients were excluded from analysis because their drug history revealed that they had taken dabigatran before admission or received dabigatran previously in other medical institutions with documented doses of dabigatran in the medical history. As a result, 131 ambulant patients and 97 inpatients were considered eligible for the re-audit analysis (Table 2). The median duration of the observation period for clinical events after the commencement of dabigatran was 197 days (range: 3–365 days). Seven ambulant patients who never revisited KIH after the start of dabigatran therapy were included in the analysis of appropriateness of dabigatran prescription but excluded from the outcome analysis.

**Table 2:** Characteristics of ambulatory patients and inpatients whose prescriptions of dabigatran were analyzed

Variables	Ambulant patients (*n* = 131)	Inpatients (*n* = 97)	*P* value
Age (years)	71 ± 9	70 ± 12	NS
Male (%)	96 (73)	64 (66)	NS
ALT (IU/L)	22 ± 12	26 ± 22	NS
AST (IU/L)	25 ± 11	29 ± 17	0.01
ALP (IU/L)	242 ± 76	238 ± 80	NS
Serum creatinine (mg/dL)	1.2 ± 0.2 [130]	1.0 ± 0.3	0.02
eGFR (mL/min/1.73 m^2^)	64 ± 14 [130]	69 ± 18	0.03
Height (cm)	162 ± 10 [47]	161 ± 10 [77]	NS
Weight (kg)	61 ± 12 [50]	60 ± 13 [80]	NS
Body surface area (m^2^)	1.6 ± 0.2 [47]	1.6 ± 0.2 [77]	NS
Numbers of concomitant medication	4.6 ± 3.5	4.2 ± 3.2	NS

Data are expressed as means ± SD. Numbers of patients whose data were available are given in brackets. Data without bracket indicate that data were available from all patients in each group. Statistical analyses were performed with the Student’s *t*-test for continuous variables and with the Chi-squared test for gender ratio

*Abbreviations*: *ALT* alanine aminotranferase, *AST* aspartate aminotransferase, *ALP* alkaline phosphatase, *NS* not significant

There were no significant differences between inpatients and ambulant patients with respect to demographic and biochemical data except for serum aspartate aminotransferase (AST) level and renal function (Table 2). While the mean serum AST level in the ambulant patients was significantly (*p* < 0.05) lower than that in inpatients, both values were within the respective normal ranges. While the ambulant patients had significantly (*p* < 0.05) lower eGFR than the inpatients (64 ± 14 vs. 69 ± 18 mL/min/1.73 m^2^, respectively), the small differences between groups (5 mL/min/1.73 m^2^) would have been clinically insignificant (Table 2). There was a good correlation between eGFR and CLcr (r = 0.72, *p* < 0.001, data are not shown) in patients whose body weights were available (50 ambulant patients and 80 inpatients). Because of inherent limitations of the retrospective study design, some demographic and biochemical data were incomplete. For instance, heights were not available for 84 out of 131 ambulant patients (64%) and 20 out of 97 inpatients (21%). Body weights were not available in 81 (62%) and 17 (18%) of ambulant patients and inpatients, respectively.

Among the 228 patients who were prescribed dabigatran for the first time during the study period, re-audit of the prescriptions showed that the prescriptions in 174 patients (76%) were appropriate because they complied with all the instructions in the prescribing information. When comparing the prescriptions for ambulant patients and inpatients, a significantly (*p* < 0.001) greater proportion of ambulant patients had inappropriate dabigatran prescriptions compared to inpatients: 33% vs 11% [odds ratio (OR): 3.8, 95% confidence interval (CI): 1.8–7.9; Table 3].

**Table 3:** Comparisons of the frequencies of inappropriate prescriptions of dabigatran between ambulant patients and inpatients

Checklist of appropriate prescriptions	Ambulant patients (*n* = 131)	Inpatients (*n* = 97)	*P* value
Overall (%)	43 (33)	11 (11)	<0.001
Unauthorized indication	0	0	NA
Violation of contraindications			
eGFR < 30 mL/min/1.73 m^2^	0 [0]	0 [0]	NA
CLCr < 30 mL/min	2 [50]	0 [80]	0.15
Concomitant use with oral itraconazole	0	0	NA
Active bleeding or hemorrhagic diathesis	0	0	NA
History of complications associated with high-risk of bleeding (cerebral hemorrhage) in the latest 6 months	0	0	NA
Concomitant dwelling of spinal or epidural catheters	0	0	NA
History of serious hypersensitivity reaction to Prazaxa®	0	0	NA
Inappropriate dose selection in reference to age			
Overdose for patients ≥ 70 years (%)	14/77 (18)	2/56 (4)	<0.05
Underdose for patients ≥ 70 years (%)	2/77 (3)	1/56 (2)	NS
Overdose for patients < 70 years (%)	0/54 (0)	0/41 (0)	NA
Underdose for patients < 70 years (%)	3/54 (6)	0/41 (0)	NS
Non-compliance with the recommendations for dose reduction			
eGFR from 30 to 50 mL/min/1.73 m^2^ (%)	2/22 (9)	1/10 (10)	NS
CLcr from 30 to 50 mL/min	0/11 (0)	1/15 (7)	NS
Past medical history of gastrointestinal bleeding (%)	1/5 (20)	3/5 (60)	NS
Concomitant use of verapamil (%)	7/10 (70)	3/7 (43)	NS
No assessment of renal function (%)	1/131 (1)	0/97 (0)	NS
PT-INR < 2.0 when dabigatran was started after discontinuation of warfarin	14/54 (26)	1/21 (5)	0.053

The figures in brackets are numbers of eligible patients

Four cases (3 ambulant patients and 1 inpatient, respectively) had more than one violations of the instructions given in the prescribing information. Statistical analyses were performed with Fisher’s exact test

*NA* not applicable, *NS* not significant

Detailed results for the re-audit of the dabigatran prescriptions are given in Table 3. There were no prescriptions for unapproved indication. There were 2 cases of violation of contraindication: 2 ambulant patients with CLcr <30 mL/min were prescribed dabigatran. No violation of contraindication was observed in inpatients. Verapamil, a strong inhibitor of P-glycoprotein, was co-administered with dabigatran without reducing the dose of dabigatran in 7 and 3 ambulant and inpatients, respectively. According to the prescribing information, a lower dose of dabigatran (110 mg twice daily) is recommended when verapamil is co-administered [1]. Five ambulant patients and one inpatient were prescribed lower doses of the drug than those recommended: 2 ambulant patients ≥ 70 years were underdosed at 75 mg once daily or 75 mg b.i.d. instead of 110 mg b.i.d. as recommended by the prescribing information and 3 ambulant patients < 70 years were underdosed at 110 mg b.i.d. instead of 150 mg b.i.d. as recommended by the prescribing information. In addition, one inpatient ≥ 70 years was underdosed at 75 mg b.i.d. instead of 110 mg b.i.d (Table 3). No appreciable reasons were found in their EMR. While the incidence of the violation of the timing for the commencement of dabigatran therapy by referring to the PT-INR of < 2.0 for the ambulant patients (26%, 14 out of 54 patients), was apparently greater than that for the inpatients (5%, 1 out of 21 patients), the difference did not reach the statistically significant level (*p* = 0.053).

The EMR documented bleeding events in 9 of 124 ambulant patients (7.3%) and in 8 of 97 inpatients (8.2%), with no significant difference in incidence between two groups (OR 0.9, 95% CI 0.3–2.3). None of the bleeding events were judged to be major according to the criteria adopted from the RE-LY study [10]. Cerebral infarction occurred in 1 of 124 ambulant patients (0.8%) and in 1 of 97 inpatients (1.0%), with no significant difference in incidence between two groups (OR 0.8, 95% CI 0.1–13). Scrutinizing their prescriptions of dabigatran, we considered that the prescription for the ambulant patient who developed cerebral infarction was appropriate, but that for the inpatient was inappropriate because he received dabigatran at 150 mg b.i.d. even though he had a past medical history of gastrointestinal bleeding. According to the prescribing information, he should have received the drug at 110 mg b.i.d. During the observation period, dose reduction of dabigatran was undertaken in 13 patients, two of whom were due to minor bleeding episodes while the others for no appreciable reasons in EMR.

## Discussion

As separation of dispensary from medical practice progresses in Japan, hospital pharmacists are getting involved in pharmaceutical care of patients in the wards than in dispensary for ambulant patients [11]. In the present study, we demonstrated that the frequency of inappropriate prescriptions of dabigatran for inpatients (11%) was significantly (*p* < 0.001) lower than that for ambulant patients (33%). Prescriptions for inpatients were audited by hospital pharmacists using abundant information available from EMR, whereas prescriptions for ambulant patients were audited by community pharmacists based on limited information mainly by questioning patients. The difference in available medical information between the two groups would have contributed largely to that in frequency of inappropriate prescriptions, provided that hospital and community pharmacists were comparable in audit competency. Thus, there is certainly advantage for hospital pharmacists to undertake audit of prescriptions for external prescriptions particularly for drugs that had contraindication or instruction of dose reduction according to renal function, age and concomitant medications. According to the annual survey of the service framework of hospital pharmacists in 2015 [11], prescription audit for ambulant patients was performed by hospital pharmacists in 46% of the hospitals surveyed in Japan.

Regarding audit competency in terms of dose adjustment according to patient’s age, there was a significantly higher frequency of inappropriate prescription [18% (14/77)] for ambulant patients aged 70 years or older: they should have been dispensed the drug at a reduced dose of 110 mg b.i.d., instead of 150 mg b.i.d., according to the prescribing information. On the contrary, only 4% (2/56) of inpatients in the same age group were given the drug at 150 mg b.i.d. (*p* < 0.05, Table 3). While we cannot categorically attribute the observed difference to that in audit competency between hospital and community pharmacists, it may be prudent to provide basic demographic information to community pharmacists for dabigatran and others of which audit needs such information.

Because an active metabolite of dabigatran etexilate, dabigatran, is eliminated almost exclusively in urine [1], renal function of patients is an important variable for individualizing dosage of the drug. In the prescribing information of dabigatran etexilate it is recommended that renal function of patients be assessed by CLcr (mL/min/body) [1]. Two ambulant patients (1.5%) with CLcr <30 mL/min were given reduced doses of dabigatran (75 mg b.i.d. or 110 mg b.i.d.) despite that dabigatran was contraindicated for these patients. One was a 97-year male weighing 54 kg and the other was a 88-year female weighing 36 kg: their renal function was 38 and 51 mL/min/1.73 m^2^ in eGFR and 24 and 28 mL/min in CLcr, respectively. In retrospect, the physicians and community pharmacists should have referred to their CLcr values. It is well known that eGFR (mL/min/1.73 m^2^) of a patient having lower body weight is tended to overestimate CLcr (mL/min/body). Because the 97-year male patient weighted 54 kg, the reason why his eGFR was greater than CLcr would have been attributed to the discordance of the Cockcroft-Gault and MDRD formulas. In contrast, no inpatients with CLcr <30 mL/min were prescribed dabigatran, probably due to the competent audit by pharmacists. Regarding the prescriptions for patients with moderately reduced renal function (CLcr 30–50 mL/min), a greater proportion of inpatients (7%) received dabigatran without appropriate dosage reduction compared to ambulant patients (0%). However, there is no significant difference between the two groups.

To our knowledge, seven previous studies [12–18] reported the frequency of potentially inappropriate dabigatran prescriptions in real-world practice in different countries (Table 4). Direct comparisons of the data obtained from the present study and those obtained from previous studies would be difficult, since all studies employed different criteria for inappropriate prescriptions. Nevertheless, the four studies [12–14, 18] that adopted criteria similar to those in the present study reported frequencies of potentially inappropriate dabigatran prescriptions (from 2.0 to 31.2%) comparable to that observed for inpatients in the present study (11%). In contrast, three other studies [15–17] reported substantially higher values (from 34.1 to 51.1%) than the present study. Two of these three studies adopted the medication appropriate index (MAI) [16, 17] for judging appropriateness of prescription. The MAI includes 10 criteria for judging appropriateness of prescription: indication, choice, dosage, modalities and practicability of administration, drug-drug interactions, drug-disease interactions, duplication, duration and cost-effectiveness, which are assessed over the whole treatment period. The remaining study [15] judged appropriateness of dabigatran prescription solely by co-administration of medications with the potential to increase bleeding risk (non-steroidal anti-inflammatory drugs, selective serotonin re-uptake inhibitors, oral corticosteroids) or P-glycoprotein inhibitors (systemic azole antifungals, macrolide antibiotics, HIV protease inhibitors, cyclosporine, dronedarone, tacrolimus, verapamil, amiodarone and quinidine). Obviously, the apparent discrepancies in frequency of inappropriate dabigatran prescriptions between the present study and these three studies would be attributable to the differences in the criteria used to judge inappropriate prescription of this drug.

**Table 4:** Summary of previous and present studies investigating inappropriate prescriptions of dabigatran

Authors [ref.]	Country	Design	Number of patients	Study patients	IM (%)	Bleeding rate (%)	Comments
Armbruster et al. [12]	USA	R	458	I	16.6	14.4	-
Simon et al. [13]	USA	R	395	A	2	16	No serum creatinine levels were available within 1 week before and after the time of dabigatran initiation in 37% of patients.
Kimmons et al. [14]	USA	R	160	I	9^a^, 10^b^	3.8	^a^Indication and ^b^dose. Only 61% of patients were newly initiated on dabigatran during the study period.
McDonald et al. [15]	USA, Canada and Australia	R	16,000	A	34.1–51.1	27.3–43.7	PIM was judged solely by co-administration of medicines potentially increase bleeding risk^c^
Larock et al. [16]	Belgium	P	69	I/A	49	14.7	MAI was used for assessing PIM
Basaran et al. [17]	Turkey	P	148	A	47	NA	MAI was used for assessing PIM
Chowdhry et al. [18]	Canada	R	109	I	31.2	NA	-
The present study	Japan	R	228	I/A	I (11) vs. A (33)	7.7	Inappropriate prescription was judged according to the descriptions in prescribing information

*R* retrospective chart review, *P* prospective study, *I* inpatients, *A* ambulant patients, *MAI* medication appropriate index, MAI is a tool designed to measure appropriateness of prescribing for people aged 65 years and older using 10 criteria comprising indication, choice, dosage, modalities and practicability of administration, drug-drug interaction and cost-effectiveness. [16, 17], *IM* inappropriate medication *PIM* potentially inappropriate medication, *NA* not available

^c^selective serotonin reuptake inhibitor, non-steroidal anti-inflammatory drug, oral corticosteroids, systemic azole antifungals, macrolide antibiotics, HIV protease inhibitors, cyclosporine, dronedarone, tacrolimus, verapamil, amiodarone and quinidine

While there are abundant reports on the frequency of dispensing error, only a few studies reported audit failure rates of pharmacists. Beex-Oosterhuis et al. [19] undertook a prospective collaborative study in 57 medical institutes to investigate the audit failure by intentionally mixing potentially inappropriate medications in routine prescriptions. Their study revealed that the pharmacists’ audit on prescriptions may overlook on average 41% of potentially inappropriate medications. In addition, Kuo et al. reported [20] that clinical pharmacists in USA identified a total of 779 cases of various types of medication errors and 58% of the errors actually reached the patients. In the present study, we found that 11% of prescriptions for dabigatran issued to inpatients in our hospital were considered inappropriate (11 of 97 inpatients). Regrettably, we were unable to determine conclusively whether an inappropriate prescription for a given inpatient was attributable either to a physicians’ error that was overlooked by pharmacists’ audit (i.e., audit failure) or to physicians’ non-acceptance of pharmacists’ inquiry about inappropriate doses of the drug.

The present study has several limitations that are inherent to a retrospective design. First, there were missing data and data collection was incomplete (Table 2). Second, comparison of the frequency of inappropriate prescriptions between ambulant patients and inpatients might have been biased, because no randomization of patients was implemented for allocating patients to the two groups. Because the present study was performed in a single hospital, its external validity is to be examined in a multi-center collaborative study with a larger number of patients. In addition, the present study is far underpowered (*n* = 228) for addressing any potential implications of pharmacists’ audit on the prevalence of thromboembolic or bleeding events in patients receiving dabigatran over a rather short observation period (the median interval of 197 days).

## Conclusion

Pharmacists’ audit of first-time prescriptions of dabigatran for inpatients using EMR may achieve better performance in eliminating inappropriate prescriptions compared to audit for ambulant patients who receive external prescriptions.
